# Comparison of Left Ventricular Functional Recovery and Remodeling After Total Thoracoscopic Mitral Valve Repair and Replacement in Patients With Mitral Regurgitation and Mildly to Moderately Reduced Left Ventricular Ejection Fraction

**DOI:** 10.1155/crp/8678425

**Published:** 2025-02-22

**Authors:** Zheng Xu, Feng Lin, Liang-Wan Chen, Xiao-Fu Dai, Zhi-Qin Lin

**Affiliations:** ^1^Department of Cardiovascular Surgery, Fujian Provincial Center for Cardiovascular Medicine, Fujian Medical University Union Hospital, Fuzhou 350001, China; ^2^Key Laboratory of Cardio-Thoracic Surgery, Fujian Medical University, Fujian Province University, Fuzhou 350001, China

**Keywords:** left ventricular function, mitral regurgitation, mitral valve repair, reduced left ventricular ejection fraction, total thoracoscopic mitral valve surgery

## Abstract

**Background:** Total thoracoscopic mitral valve surgery (TT-MVS) is a minimally invasive technique for mitral regurgitation (MR), but its impact on left ventricular (LV) function and remodeling in patients with reduced LV ejection fraction (LVEF) is unclear.

**Methods:** We retrospectively compared 94 patients who underwent total thoracoscopic mitral valve repair (TT-MVr) or total thoracoscopic mitral valve replacement (TT-MVR) for MR and reduced LVEF at our center from January 1, 2017, to December 31, 2022. We assessed LV functional recovery and remodeling by echocardiography at baseline, 1 week, 3 months, and 6 months after surgery.

**Results:** A total of 43 patients underwent TT-MVr and 51 patients underwent TT-MVR. Both groups had similar early outcomes, hospital mortality, and postoperative complications. The TT-MVr group had higher LVEF and lower left ventricular end-diastolic diameter (LVEDD) and left ventricular end-systolic diameter (LVESD) than the TT-MVR group at 3 and 6 months after surgery (*p* < 0.05 for all comparisons). Both groups improved in New York Heart Association (NYHA) functional class from baseline to 6 months after surgery (*p* < 0.05 for all comparisons).

**Conclusion:** TT-MVr and TT-MVR are feasible and safe for patients with MR and reduced LVEF, but TT-MVr is associated with better LV functional recovery and remodeling within 6 months after surgery. TT-MVr should be preferred over TT-MVR whenever possible in this high-risk population. Further studies are needed to evaluate the long-term outcomes of TT-MVS in this population.

## 1. Introduction

Mitral regurgitation (MR) is the most prevalent form of mitral valve disease, affecting about 2% of the general population [1]. MR leads to left ventricular (LV) volume overload, pulmonary congestion, reduced cardiac output, and eventually LV dysfunction and remodeling, which impair cardiac performance and quality of life [2, 3]. Surgical intervention is indicated for patients with severe symptomatic MR or asymptomatic MR with evidence of LV dysfunction or dilation [4]. Mitral valve repair (MVr) is preferred over mitral valve replacement (MVR) whenever feasible, as it preserves the native valve and subvalvular apparatus, reduces the risk of thromboembolism and infection, and improves long-term survival and quality of life [5]. However, the choice between MVr and MVR is not always straightforward, as some patients may have complex valve pathology, high surgical risk, or contraindications for valve repair.

However, conventional mitral valve surgery (MVS) requires a median sternotomy or a mini-thoracotomy, which are associated with significant morbidity, prolonged hospitalization, and delayed recovery. To overcome these limitations, minimally invasive techniques have been developed to perform MVS through small incisions in the right side of the chest using thoracoscopic or robotic assistance [6]. These techniques offer several advantages over conventional MVS, such as reduced trauma, bleeding, pain, infection, hospital stay, and postoperative complications; improved cosmetic results; and faster return to normal activities. Among the minimally invasive techniques for MVS, total thoracoscopic mitral valve surgery (TT-MVS) has emerged as a promising option that combines the benefits of minimal invasiveness with the precision and dexterity of direct vision and manual suturing [7].

Previous studies have mainly focused on the feasibility, safety, and short-term outcomes of TT-MVS [8], but there is limited evidence on the impact of TT-MVS on LV function and remodeling in patients with MR and reduced left ventricular ejection fraction (LVEF), which is a high-risk subgroup with poor prognosis. Therefore, we conducted a retrospective study of a case series of 94 consecutive patients who underwent TT-MVr or TT-MVR for MR and reduced LVEF at our center between January 2017 and December 2022. The aim of this study was to compare the outcomes of TT-MVr and TT-MVR in terms of LV functional recovery and remodeling within 6 months after surgery. We hypothesized that TT-MVr would be superior to TT-MVR in terms of LV functional recovery and remodeling in this high-risk population.

## 2. Methods

### 2.1. Study Patients and Data Collection

This study is a retrospective analysis of a prospectively collected sample database. Two hundred and seventy consecutive symptomatic patients with at least moderate or severe MR and reduced LVEF (defined as EF < 50% in this study) underwent TT-MVr or TT-MVR at our institution between January 1, 2017, and December 31, 2022. All patients who received TT-MVr or TT-MVR during this period at our institution are included in the study. The present study was approved by the Ethics Committee of Union Hospital, Fujian Medical University (Ethics approval number: 2023ZH234), and fully adhered to the principles of the Declaration of Helsinki. The patient(s) or guardian(s) provided consent for the publication of all images, clinical data, and relevant information. Due to the retrospective nature of the study, written informed consent was waived. The exclusion criteria were as follows: (1) patients with greater than moderate mitral stenosis, active endocarditis, constrictive pericarditis, or any other concomitant cardiac surgical procedures (e.g., tricuspid valve repair or replacement, coronary artery bypass, or radiofrequency ablation of atrial fibrillation); (2) patients with acute MR due to myocardial infarction, infective endocarditis, or iatrogenic causes; and (3) patients with prior cardiac surgical procedure. Of the 270 patients who underwent TT-MVS for MR and reduced LVEF during the study period, 176 patients were excluded for the following reasons: 58 patients had concomitant tricuspid valve surgery, 49 patients had radiofrequency ablation for atrial fibrillation, 32 patients had prior cardiac surgery, 18 patients had acute MR due to myocardial infarction, 11 patients had infective endocarditis, and 8 patients had simultaneous coronary artery bypass grafting. The remaining 94 patients met the inclusion and exclusion criteria and were included in the final analysis.

The decision to perform valve repair or replacement was made intraoperatively by the surgeon based on the feasibility of repair, which depended on factors such as valve morphology, etiology, severity, and mechanism of MR.

Patient characteristics, laboratory values, echocardiographic results, and in-hospital and follow-up outcomes were recorded at the time of presentation. Transthoracic echocardiography examinations were routinely performed to identify the mechanism and etiology of MR before surgery. Quantification of MR was evaluated semi-quantitatively by color Doppler imaging at transthoracic echocardiography and was graded as follows: 1 = mild, 2 = moderate, 3 = moderately severe, and 4 = severe [9].

The primary outcomes of interest were LV functional recovery and remodeling within 6 months after surgery. LV functional recovery was assessed using two methods: (1) LVEF calculated using the Simpson method, and (2) global longitudinal peak strain (GLPS) measured by 2D speckle-tracking echocardiography, with the GLPS value calculated as the average of strain values from both 4-chamber and 2-chamber views. Cardiac function was assessed with the New York Heart Association (NYHA) functional class at baseline, 1 week, 3 months, and 6 months after both procedures. LV remodeling was estimated by left ventricular end-diastolic diameter (LVEDD), left ventricular end-systolic diameter (LVESD), end-diastolic volume index (EDVI), and end-systolic volume index (ESVI), with EDVI and ESVI calculated using the modified Simpson's rule and indexed to body surface area, at baseline, 1 week, 3 months, and 6 months after both procedures. The secondary outcomes of interest were postoperative complications and mortality after surgery.

### 2.2. Surgical Procedures and Postsurgical Treatment

The choice of surgical technique was based on the surgeon's preference and the patient's suitability for valve repair. Both procedures were initially performed under general anesthesia with hypothermic cardiopulmonary bypass (CPB). A transesophageal echocardiography (TEE) probe was inserted to monitor the cardiac function and the mitral valve anatomy and function throughout the surgery. To drain the vena cava blood, two central venous catheters were inserted: one through the right internal jugular vein and another through the right femoral vein. The right femoral artery was used for arterial cannulation during CPB. The surgery was performed by experienced surgeons at our center using total thoracoscopic technology. The right chest had two small incisions in the fourth intercostal space: a thoracoscope camera and a Chitwood cross-clamp went through a 1-cm incision at the axillary midline level, and a 3-cm anterolateral mini-thoracotomy incision served as the main working port. The valve morphology was assessed and the repair technique was selected according to Carpentier's classification [10]. The repair techniques included annuloplasty with a ring, leaflet resection, chordal shortening or transfer, cleft suture, decalcification, commissure plasty, and neochordae implantation. In case of intraoperative failure of TT-MVr, such as residual MR, valve dysfunction, or ring dehiscence, conversion to TT-MVR was performed as a salvage procedure. The replacement techniques included either mechanical or bioprosthetic valves with preservation of subvalvular apparatus whenever possible. In both groups, hemostasis was secured and chest tubes were placed. The incisions were closed with absorbable sutures. The patients were transferred to the intensive care unit (ICU) for postoperative care. Both groups received similar postoperative care. Patients with a mechanical valve needed warfarin therapy for life, while those with a biological valve or a ring only needed it for 3 months. Other drugs such as angiotensin-converting enzyme inhibitors, *β*-adrenoreceptor blockers, and aldosterone antagonists were routinely used to reverse myocardial remodeling after surgery.

### 2.3. Statistical Analysis

We expressed continuous variables as mean ± standard deviation or median (interquartile range (IQR)), depending on their distribution, and compared them using Student's *t*-test or Wilcoxon signed-rank test, respectively. We presented categorical variables as counts or percentages and compared them using chi-squared test or Fisher's exact test, as appropriate. To compare LV function recovery and remodeling outcomes across different time points and to assess the effect of surgical type, we fitted two types of generalized linear mixed-effects models for repeated-measures analysis. For NYHA functional class, we used a multinomial logistic model with an ordinal response variable. For LVEF, GLPS, EDVI, ESVI, LVEDD, and LVESD outcomes, we used a Gaussian model with an identity link function. We treated differences among patients as a random effect and added “group × time” to the main effects with adjustment for baseline covariates. We excluded patients with missing data for a particular variable from the analysis of that variable. We fitted the models using the glmer function in the lme4 package and the clmm function in the ordinal package in R. This methodology is consistent with our previous studies, including one on coronary artery disease where we established a robust statistical approach for evaluating cardiac function recovery [11] and another on aortic valve replacement where we compared postoperative pain and quality of life between total thoracoscopic surgery and conventional full sternotomy [12]. We considered a two-sided *p* value of < 0.05 as statistically significant. We performed all analyses using SPSS v.26.0 (IBM SPSS Inc., Armonk, NY) and R 4.3.0.

## 3. Results

### 3.1. Baseline Characteristics

A total of 94 cases were included in this study, 43 patients for TT-MVr and 51 patients for TT-MVR. The patients' baseline characteristics and comorbidities are shown in Table 1. There were no significant differences between the two groups in terms of baseline demographic characteristics and comorbidities. The mean age of all patients was 56 (48.25–64) years, and 50.0% were male. The median LVEF for the included patients was 44.5% (IQR: 41.9%–46.5%), and the cohort included 18 patients with LVEF below 40% but greater than 30%. A total of 85 (90.4%) patients had NYHA class III or IV. The main cause of MR was ruptured chordae tendineae in 35 patients, followed by myxomatous mitral valve disease in 26, degenerative valves in 19, endocarditis in 9, and rheumatic disease in 5. Among the 9 patients with endocarditis, 7 had healed endocarditis with residual MR and 2 had nonactive endocarditis with moderate MR. Two patients (4.7%) in the TT-MVr group required conversion to TT-MVR due to intraoperative failure of repair. Both cases had severe MR with posterior leaflet prolapse and ruptured chordae tendineae.

### 3.2. Operative Data and Early Outcomes

Most patients (88.4%) who underwent TT-MVr received complete ring annuloplasty, while the rest (11.6%) did not use a ring. For valve replacement, mechanical valves were used in 70.6% of cases and bioprostheses in 29.4%. Subvalvular apparatus preservation was performed in 39 patients and omitted in 12.

Table 2 shows the operative data and early outcomes of the study participants. The TT-MVr group had significantly longer operation duration (TT-MVr: 222 (205.5–247) min vs. TT-MVR: 205 (194–221.5) min, *p*=0.004), CPB time (TT-MVr: 123 (114–130) min vs. TT-MVR: 103 (95.5–113) min, *p* < 0.001), and aortic cross-clamp (ACC) time (TT-MVr: 87 (79–94.5) min vs. TT-MVR: 63 (56–64) min, *p* < 0.001) than the TT-MVR group. However, there were no significant differences between the two groups on ICU stay, hospital stay, hospital mortality, or any of the early complications. The overall hospital mortality was 2.1%, with one death (due to severe pneumonia) in the TT-MVr group and one death (due to low cardiac output syndrome) in the TT-MVR group (*p*=0.903). At 6 months postoperatively, most patients in the TT-MVr group (88.4%, 38/43) had no or mild MR, while a few had moderate (10.5%, 4/43), moderately severe (2.3%, 1/43), or severe (0%, 0/43) MR. Similarly, most patients in the TT-MVR group (98.0%, 50/51) had no or trivial valve dysfunction or paravalvular leak, while one patient (2.0%, 1/51) had moderate paravalvular leak and none (0%, 0/51) had severe valve dysfunction or paravalvular leak.

### 3.3. Change in LV Function Indicators and NYHA Functional Class

**Figure 1:**
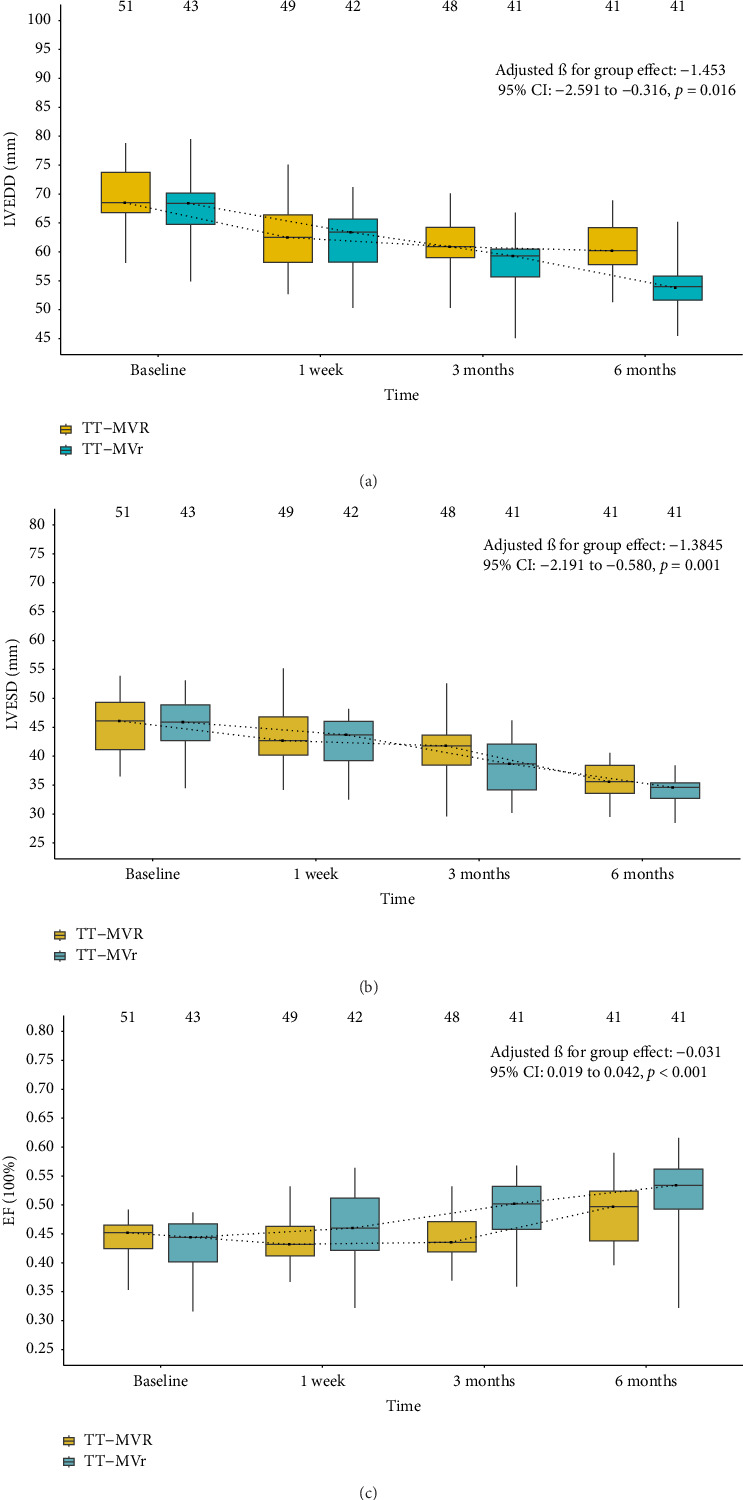
Median (interquartile range) of LVEF, GLPS, EDVI, ESVI, LVEDD, and LVESD of the two groups at baseline and follow-up. EDVI, end-diastolic volume index; ESVI, end-systolic volume index; LVEDD, left ventricular end-diastolic diameter; LVEF, left ventricular ejection fraction; LVESD, left ventricular end-systolic diameter; GLPS, global longitudinal peak strain; TT-MVr, total thoracoscopic mitral valve repair; TT-MVR, total thoracoscopic mitral valve replacement.

**Figure 2:**
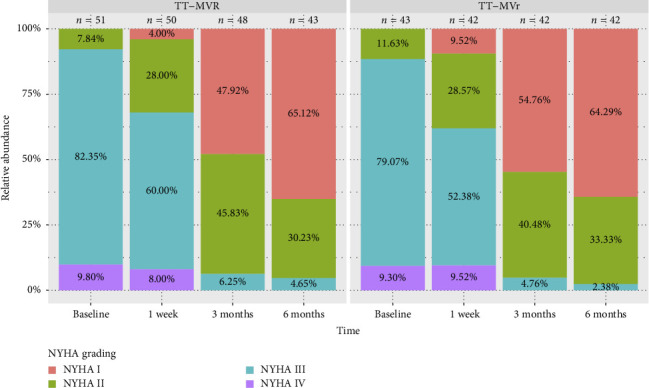
Comparison of the proportion of patients in the different subclasses of NYHA function of the two groups at baseline and follow-up. NYHA, New York Heart Association; TT-MVr, total thoracoscopic mitral valve repair; TT-MVR, total thoracoscopic mitral valve replacement.

Figure 1 demonstrates that the surgical strategy (TT-MVr or TT-MVR) significantly influenced the outcomes of LVEF, GLPS, EDVI, ESVI, LVEDD, and LVESD using mixed-model analysis.  Table 3 summarizes the mixed-model repeated measures analysis for LV function indicators and NYHA functional class. There were no significant differences between the two groups in terms of GLPS, ESVI, LVEDD, and LVESD at baseline and week 1. However, at months 3 and 6, the TT-MVr group had significantly higher GLPS and lower ESVI, LVEDD, and LVESD compared to the TT-MVR group (*p*=0.001 and *p* < 0.001 for GLPS; *p*=0.047 and *p* < 0.001 for LVEDD; *p* < 0.001 and *p*=0.016 for LVESD; *p*=0.012 and *p* < 0.001 for ESVI, respectively). Similarly, there were no significant differences in LVEF and EDVI at baseline between the two groups. However, at week 1, month 3, and month 6, the TT-MVr group had significantly higher LVEF and lower EDVI than the TT-MVR group (*p*=0.005, *p* < 0.001, and *p* < 0.001 for LVEF; *p*=0.034, *p* < 0.001, and *p* < 0.001 for EDVI, respectively). As shown in Figure 2, there were no significant differences between the TT-MVr and TT-MVR groups in terms of NYHA class (adjusted *β* for group effect: −0.224, 95% CI: −0.708 to 0.260, *p*=0.364). Both groups showed significant improvement in NYHA class from baseline to week 1, month 3, and month 6 (*p* < 0.05 for all comparisons).

## 4. Discussion

In this study, we compared the outcomes of TT-MVr and TT-MVR in terms of LV functional recovery and remodeling within 6 months after surgery in patients with MR and reduced LVEF. The main findings of this study are as follows: (1) TT-MVr and TT-MVR are both feasible and safe techniques for patients with MR and reduced LVEF, with comparable early outcomes and low mortality rates; (2) TT-MVr is associated with better LV functional recovery and remodeling than TT-MVR at 6 months after surgery; and (3) both groups show similar improvement in NYHA functional class.

Previous studies have shown that MVS can improve LV function and reduce heart failure severity in patients with MR and reduced LVEF [13, 14]. However, research by Pavan Atluri, MD, and colleagues focused on a right thoracotomy approach in patients with LVEF ≤ 40%, while our study specifically examined total thoracoscopic procedures in patients with LVEF < 50%. Similarly, Zainab Samad and collaborators concentrated on a subgroup of patients with functional MR and severe LV dysfunction (LVEF ≤ 30%), whereas our study focused on patients with moderately reduced LVEF (30%–50%). These differences highlight the unique aspects and contributions of our research. MVr can restore normal valve function while preserving the subvalvular apparatus, annulus, and LV geometry. In contrast, MVR requires chordal excision and enlargement of the mitral annulus, which may negatively impact LV geometry, contractility, and systolic function despite correcting the MR. This disadvantage in LV function with MVR compared with MVr is accentuated in patients with pre-existing LV dysfunction [15]. In our study, we focused on a high-risk subgroup of patients with MR and reduced LVEF who underwent TT-MVS, which is a minimally invasive technique that offers several advantages over conventional MV surgery. However, we encountered two cases of conversion from TT-MVr to TT-MVR due to intraoperative failure of repair, which suggests a potential risk and challenge of performing TT-MVr in patients with complex valve pathology.

Our results showed that both TT-MVr and TT-MVR were feasible and safe procedures with low rates of hospital mortality and postoperative complications. The TT-MVr group had significantly longer operation duration, CPB time, and ACC time than the TT-MVR group, which could be explained by the technical complexity and learning curve of the repair technique [16]. However, these differences did not translate into significant differences in ICU stay or hospital stay between the two groups. This finding implies that the advantages of minimally invasive surgery, such as reduced trauma, bleeding, pain, infection, and postoperative complications, may compensate for the risks of prolonged CPB time and operation time. Nevertheless, CPB time and operation time remain important factors that may affect the outcomes of cardiac surgery [17]. Therefore, these factors should be optimized and their adverse effects on patients should be minimized.

Our study also showed that TT-MVr was associated with better LV functional recovery and remodeling than TT-MVR within 6 months after surgery. This finding is consistent with previous studies that have reported that MVr may prevent or delay the progression of LV dilation and dysfunction by preserving the mitral annular dynamics and synchrony, while MVR may impair these functions by implanting a prosthetic valve [18]. Moreover, MVr may reduce the risk of thromboembolism and infection associated with prosthetic valves, which may also affect LV function [19]. Furthermore, MVr reduces ventricular afterload more than MVR by avoiding prosthesis–patient mismatch [20].

In terms of functional status, we also found that both TT-MVr and TT-MVR groups showed significant improvement in NYHA functional class from baseline to 6 months after surgery. This finding indicates that both surgical techniques can relieve the symptoms of heart failure and improve the quality of life of patients with MR and reduced LVEF. However, we did not find any significant difference between the two groups in terms of NYHA functional class. This finding may seem contradictory to the finding of better LV function recovery in the TT-MVr group. One possible explanation for this finding is that NYHA functional class is a subjective measure of heart failure symptoms, which may be influenced by other factors such as comorbidities, medication adherence, or patient expectations. Moreover, NYHA functional class may not be sensitive enough to detect subtle differences in LV function between the two groups. Therefore, NYHA functional class may not reflect the true impact of TT-MVr and TT-MVR on LV function and remodeling in patients with MR and reduced LVEF [21]. Moreover, the follow-up duration of 6 months may not be long enough to observe the full impact of surgical technique on functional status, as LV function and remodeling may continue to evolve over time.

This study demonstrated that most patients had no or trivial residual MR or valve dysfunction or paravalvular leak at 6 months postoperatively, suggesting a satisfactory procedural outcome and valve function. These results are in line with previous studies that showed beneficial effects of mitral valve repair or replacement for MR. However, some patients had moderate or moderately severe residual MR or paravalvular leak after surgery, which could compromise LV function recovery and increase the risk of heart failure and mortality [22, 23]. Further research is needed to investigate the impact of moderate or moderately severe residual MR or paravalvular leak on LV function recovery.

The choice of intervention between repair and replacement may also affect the long-term outcome of patients with MR and reduced LVEF. Although both repair and replacement can reduce MR severity and improve LV function, repair may have some advantages over replacement in terms of preserving native valve structure and function, reducing thromboembolic and bleeding events, and improving survival. However, repair may also have some limitations such as higher recurrence rate of MR, technical complexity, and variability in surgical skills. Therefore, the decision between repair and replacement should be individualized based on the patient's characteristics, valve anatomy, surgical risk, and expected benefit.

It is worth emphasizing that the choice of surgical technique for TT-MVS is influenced by several factors, such as the etiology, mechanism, severity, and feasibility of MR; the patient's age, comorbidities, preferences, and life expectancy; and the surgeon's experience, preference, and skill. TT-MVr requires more technical skill and experience than TT-MVR, as it involves complex repair techniques using total thoracoscopic technology such as leaflet resection, chordal shortening or transfer, commissure plasty, and neochordae implantation [24]. The learning curve for TT-MVr is steep and requires adequate training and supervision [25]. Moreover, the surgeon's preference and confidence in performing TT-MVr may affect the decision-making process and the outcomes of the procedure.

Our study has several limitations. First, it was a retrospective study with a relatively small sample size and a single-center design, which may limit the generalizability of our findings. Moreover, the nonrandomized nature of our study may introduce selection bias and further limit the generalizability of our results. Future randomized controlled trials are needed to validate our findings. Second, we did not have data on long-term outcomes such as survival, recurrence of MR, or need for reoperation. Third, we did not perform quantitative analysis of valve function and LV function using intraoperative TEE before and after surgery. Fourth, we assessed LV systolic function using LVEF, GLPS, EDVI, ESVI, LVEDD, and LVESD. However, we acknowledge that our dataset still lacks myocardial work and data related to left atrial volume and function, which limits the scope of this study. Future research should incorporate these advanced measurements to provide a more nuanced assessment of both ventricular and atrial performance and remodeling [26]. Fifth, we did not have data on the quality of life, functional status, or cost-effectiveness of the two surgical techniques, which are important outcomes for patients and healthcare providers.

## 5. Conclusion

In conclusion, our study suggests that TT-MVr and TT-MVR are both feasible and safe techniques for patients with MR and reduced LVEF, with comparable early outcomes and low mortality rates. However, TT-MVr is associated with better LV functional recovery and remodeling than TT-MVR within 6 months after surgery. Therefore, TT-MVr should be preferred over TT-MVR whenever possible in this high-risk population. Further studies are needed to confirm our findings and to evaluate the long-term outcomes of TT-MVS in this high-risk population.

## Figures and Tables

**Table 1:** Comparison of patients' baseline demographic and clinical characteristics.

Variables^a^	Total sample (*n* = 94)	Patient groups		
		TT-MVr (*n* = 43)	TT-MVR (*n* = 51)	*p* value
Age (yr)	56 (48.25–64)	54 (47.5–61)	58 (49–65)	0.128
Male (*n*)	47	26	21	0.098
BMI (kg/m^2^)	21.24 (18.41–24.5375)	21.54 (18.41–21.24)	21.11 (18.415–24.165)	0.278
Smoking history (*n*)	25	9	16	0.363
Diabetes (*n*)	14	7	7	0.956
Hypertension (*n*)	16	4	12	0.120
CAD(*n*)	15	9	6	0.354
Prior MI (*n*)	7	4	3	0.529
COPD (*n*)	7	4	3	0.529
Liver dysfunction (*n*)	8	5	3	0.320
Dialysis (*n*)	9	4	5	0.934
Peripheral vascular disease	4	1	3	0.395
Cancer history (*n*)	7	3	4	0.873
Stroke history (*n*)	6	4	2	0.288
History of AF (*n*)	14	5	9	0.599
NYHA class (*n*)				
II	9	5	4	0.925
III	76	34	42	
IV	9	4	5	
Preoperative echocardiographic data				
LVEDD (mm)	68.5 (65.9–72.025)	68.4 (64.75–70.15)	68.5 (66.8–73.75)	0.253
LVESD (mm)	46.0 (42.125–49.2)	45.9 (42.7–48.9)	46.1 (41.15–49.3)	0.982
LVEF (%)	44.5 (41.9–46.5)	44.4 (40.15–46.75)	45.2 (42.45–46.5)	0.414

Abbreviations: AF, atrial fibrillation; BMI, body mass index; CAD, coronary artery disease; COPD, chronic obstructive pulmonary disease; LVEDD, left ventricular end-diastolic diameter; LVEF, left ventricular ejection fraction; LVESD, left ventricular end-systolic diameter; MI, myocardial infarction; NYHA, New York Heart Association; TT-MVr, total thoracoscopic mitral valve repair; TT-MVR, total thoracoscopic mitral valve replacement.

^a^Nonnormally distributed variables are presented as the median (interquartile range (IQR)) and categorical data as number.

**Table 2:** Operative data and postoperative in-hospital outcomes.

Variables^a^	Total sample (*n* = 94)	Patient groups		
		TT-MVr (*n* = 43)	TT-MVR (*n* = 51)	*p* value
Operation duration (minutes)	213 (197–227.75)	222 (205.5–247)	205 (194–221.5)	0.004
CPB time (minutes)	113 (102–125)	123 (114–130)	103 (95.5–113)	< 0.001
ACC time (minutes)	67.5 (60.5–87)	87 (79–94.5)	63 (56–64)	< 0.001
Intensive care unit stay (days)	2 (1–3)	2 (1–3)	2 (1–3)	< 0.805
Hospital stay (days)	10 (9–11)	9 (8–11)	10 (9–11.5)	< 0.136
Hospital mortality	2.1%	2.3%	2.0%	0.903
Early complications				
Respiratory complication (*n*)	20	8	12	0.743
Prolonged ventilation (*n*)	6	2	4	0.528
LCOS requiring MCS (*n*)	3	2	1	0.460
Cardiocerebral events (*n*)	3	2	1	0.460

Abbreviations: ACC, aortic cross-clamp; CPB, cardiopulmonary bypass; LCOS: low cardiac output syndrome; MCS: mechanical cardiac support; TT-MVr, total thoracoscopic mitral valve repair; TT-MVR, total thoracoscopic mitral valve replacement.

^a^Nonnormally distributed variables are presented as the median (interquartile range (IQR)) and categorical data as number.

**Table 3:** Summary of mixed-model repeated measures analysis for left ventricular function indicators and NYHA class.

Response variables	TT-MVr		TT-MVR		Difference (95% CI; *p* value)
	*n*	Adjusted mean	*n*	Adjusted mean	
LVEF^a^					
Baseline	43	0.431	51	0.436	0.005 (−0.015∼0.025; *p*=0.617)
Week 1	42	0.464	49	0.435	−0.029 (−0.049∼−0.009; *p*=0.005)
Month 3	41	0.497	48	0.440	−0.057 (−0.078∼−0.037; *p* < 0.001)
Month 6	41	0.529	41	0.484	−0.044 (−0.065∼−0.023; *p* < 0.001)
GLPS^b^					
Baseline	43	−14.2	51	−14.2	−0.003 (−0.251∼0.246; *p*=0.983)
Week 1	42	−13.4	49	−13.6	−0.249 (−0.501∼0.003; *p*=0.053)
Month 3	41	−15.6	48	−15.1	0.418 (0.164∼0.672; *p*=0.001)
Month 6	41	−17.5	41	−17.7	0.905 (0.642∼1.167; *p* < 0.001)
EDVI^c^					
Baseline	43	86.4	51	86.4	−0.031 (−0.339∼0.277; *p*=0.843)
Week 1	42	84.9	49	85.2	0.338 (0.026∼0.650; *p*=0.034)
Month 3	41	83.3	48	84.3	0.973 (0.659∼1.287; *p* < 0.001)
Month 6	41	82.0	41	83.5	1.442 (1.121∼1.764; *p* < 0.001)
ESVI^d^					
Baseline	43	54.5	51	54.8	0.347 (−0.326∼1.021; *p*=0.312)
Week 1	42	52.2	49	52.2	−0.037 (−0.721∼0.648; *p*=0.916)
Month 3	41	48.5	48	49.3	0.888 (0.197∼1.578; *p*=0.012)
Month 6	41	45.5	41	47.8	2.309 (1.594∼3.023; *p* < 0.001)
LVEDD^e^					
Baseline	43	69.2	51	69.7	0.500 (−0.961∼1.961; *p*=0.502)
Week 1	42	63.2	49	62.6	−0.675 (−2.167∼0.816; *p*=0.375)
Month 3	41	59.5	48	61.0	1.526 (0.021∼3.031; *p*=0.047)
Month 6	41	55.3	41	60.0	4.799 (3.246∼6.351; *p* < 0.001)
LVESD^f^					
Baseline	43	45.6	51	45.7	0.063 (−1.037∼1.160; *p*=0.910)
Week 1	42	42.5	49	43.2	0.671 (−0.454∼1.800; *p*=0.242)
Month 3	41	38.6	48	41.8	3.245 (2.109∼4.380; *p* < 0.001)
Month 6	41	34.0	41	35.4	1.446 (0.271∼2.620; *p*=0.016)
NYHA class^g^					
Baseline	43	1.463	51	1.645	0.183 (−0.726∼1.091; *p*=0.694)
Week 1	42	0.113	50	0.474	0.362 (−0.548∼1.271; *p*=0.436)
Month 3	42	−3.347	48	−3.033	0.314 (−0.542∼1.169; *p*=0.472)
Month 6	42	−3.788	43	−3.772	0.016 (−0.917∼0.948; *p*=0.972)

Abbreviations: CI, confidence interval; EDVI, end-diastolic volume index; ESVI, end-systolic volume index; GLPS, global longitudinal peak strain; LVEDD, left ventricular end-diastolic diameter; LVEF, left ventricular ejection fraction; LVESD, left ventricular end-systolic diameter; NYHA, New York Heart Association; TT-MVr, total thoracoscopic mitral valve repair; TT-MVR, total thoracoscopic mitral valve replacement.

^a^Adjusted for baseline covariates in a Gaussian model with an identity link function: age, gender, AF, and baseline LVEF.

^b^Adjusted for baseline covariates in a Gaussian model with an identity link function: age, gender, AF, and baseline GLPS.

^c^Adjusted for baseline covariates in a Gaussian model with an identity link function: age, gender, AF, and baseline EDVI.

^d^Adjusted for baseline covariates in a Gaussian model with an identity link function: age, gender, AF, and baseline ESVI.

^e^Adjusted for baseline covariates in a Gaussian model with an identity link function: age, gender, AF, and baseline LVEDD.

^f^Adjusted for baseline covariates in a Gaussian model with an identity link function: age, gender, AF, and baseline LVESD.

^g^Adjusted for baseline covariates in a multinomial logistic model with an ordinal response variable: age, gender, and baseline NYHA class.

## Data Availability

The data that support the findings of this study are available on request from the corresponding authors. The data are not publicly available due to privacy or ethical restrictions.
